# Tribological performance of electrically conductive and self-lubricating polypropylene–ionic-liquid composites†

**DOI:** 10.1039/d3ra00712j

**Published:** 2023-03-10

**Authors:** Samuel Franz Gatti, Felix Gatti, Tobias Amann, Andreas Kailer, Kevin Moser, Patrick Weiss, Claudia Seidel, Jürgen Rühe

**Affiliations:** a Fraunhofer Institute for Mechanics of Materials IWM, MicroTribology Center μTC Woehlerstraße 11 79108 Freiburg Germany felix.gatti@iwm.fraunhofer.de; b Fraunhofer Cluster of Excellence Programmable Materials CPM Woehlerstraße 11 79108 Freiburg Germany; c Fraunhofer Institute for Chemical Technology ICT Joseph-von-Fraunhofer-Str. 7 76327 Pfinztal Germany; d University Freiburg, IMTEK – Department of Microsystems Engineering Georges-Koehler-Allee 103 79110 Freiburg Germany

## Abstract

In this work, self-lubricating and electrically conductive polymers on a polypropylene (PP) matrix were prepared and investigated. These properties were obtained by additivating PP with carbon black (CB) and multi-walled carbon nanotubes (MWCNTs), in combination with a surface active ionic liquid (IL, trihexyltetradecylphosphonium docusate [P_66614_][DOC]). These polymeric composites are expected to achieve coefficients of friction (COFs) comparable to lubricated systems. Combined with electrical conductivity, these materials could be applied in electrically loaded tribosystems. The COF was reduced by up to 25% compared to that of plain PP, and high electrical conductivity and self-lubrication were achieved. Fundamental differences between the carbon-based fillers in their interaction with IL were investigated with high-resolution surface analysis (TEM, AFM) and Raman and ATR-FTIR spectroscopy. By varying the tribological test parameters, the application limits of self-lubrication were identified. It was demonstrated that the contact pressure has a strong influence on the COF. Therefore, this work points to potential applications in (*e.g.* 3D-printed) bearings and electrically loaded bearings where electrical conductivity and relatively low COFs are required.

## Abstract 2

Improved friction and electrical conductivity through self-lubricating polypropylene composites with embedded ionic liquids and carbon-based fillers.

## Graphical abstract

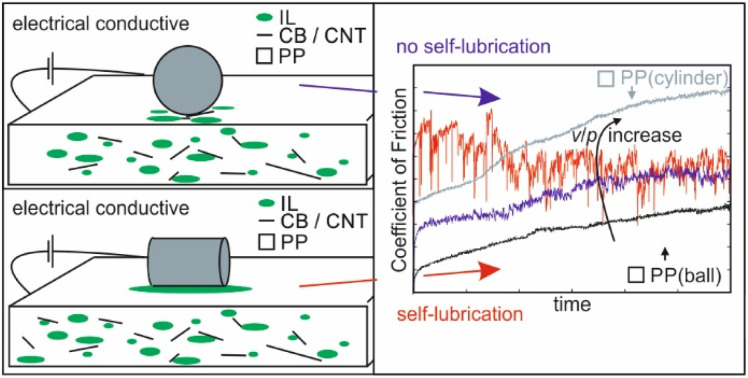

## 1. Introduction

Friction is responsible for 23–30% of worldwide energy consumption and is therefore one of the largest energy consumers in the world.^1,2^ Due to this, reduction of friction is key for energy efficiency and sustainability of mechanical devices, which is achieved in conventional systems by the usage of lubricants. For liquid lubrication, influences of viscosity, sliding velocity and contact pressure or load are well described by the Stribeck curve. This results in a minimum coefficient of friction (COF) in the hydrodynamic or elastohydrodynamic lubrication range, which is related to the viscosity of the lubricant. Under these parameters a stable tribofilm is formed.^3^ However, a unified picture is still lacking for self-lubricating systems. The tribological processes (load spectrum) that lead to the release of the lubricant (solid, liquid) from the polymer compound and the resulting interactions of the lubricant with the sliding surface are not yet fully understood.

This work focuses on polymer-based composites, enabling self-lubrication and electric conductivity by adding carbon-based additives and ionic liquids (ILs) with the aim of shedding light on the influence of testing parameters as described by the Stribeck curve in liquid lubricant systems. This approach intends to meet two challenges posed to tribological systems. Electrical discharges that can occur in various applications (*e.g.* leakage currents in electric motors in automobiles) are to be dissipated by the electrical conductivity of the material. This prevents the formation of high voltages, which could damage the material and the lubricant (*e.g.* electro-pitting, decomposition). In addition, the self-lubrication of the material should make the application of an external lubricant obsolete. However, influences of testing parameters still need to be described. In the following, important results for this work shall be discussed.

In the context of tribological application, certain polymers are gaining an increasing importance for technical applications due to their variability of properties by adding additives as well as their accessibility and workability.^4,5^ Another big advantage of polymers, for example polypropylene (PP) is their low cost.^6^ Limiting factors for polymers in terms of their tribological load capacity and working range and their temperature stability at high sliding velocities and contact pressures due to the low heat dissipation in comparison to ceramics.^6^ Without the addition of additives, polymers generally show weak tribological performance. By adding additives to a polymeric matrix-material, mechanical, chemical and tribological properties of the composite are improved.^7–10^ Properties of particular interest are hardness, electrical conductivity, COF and wear.

Recently, liquid lubricants based on ionic liquids (ILs) have been gaining interest as lubricants or additives due to their unique properties.^11–14^ Depending on their chemical structure, certain ILs exhibit ionic conductivity, good thermal stability, non-flammability and negligible volatility. Good tribological behavior of some ILs is a result of chemical interaction with the surface (tribofilm formation).^15^ The stability of an IL tribofilm depends on the interaction of the anions with the surface. Since it is mainly these surface interactions that influence tribological behavior, certain ILs are promising additives.^16^ In addition, the coulombic attraction between the surface and the IL influences the adsorption of the ions. Thus, it is possible to reversibly influence the COF by changing the surface charges.^17–19^ This behavior makes it possible to electrochemically control friction in order to adapt the COF to the load spectrum.^15,20^ Various different mechanisms are known for the formation of surface charges, depending on the interacting surfaces and lubricants.^17,21–23^ The influence of H_2_O and CO_2_ content, shear rates, temperature and pressure or load on mechanical and tribological properties have been discussed in the literature.^19,24–28^ Changes in tribological properties when ILs are used are attributed to changes in local structures or surface interactions.^19,27^

Carbon-based additives like graphene, carbons with a lack of long-distance ordering like carbon black (CB), as well as single- and multi-walled carbon nanotubes (CNTs and MWCNTs) are of particular interest in tribological applications. Their use as polymer additives influences the hardness, wear resistance and the materials electrical properties. In combination with ILs, synergistic effects for reducing friction and wear have also been demonstrated in tribological tests.^29–36^ The electrical conductivity of a polymer compound is highly depending on the homogeneity of the distribution of the conducting filler (especially CB and (MW)CNTs) throughout the matrix, which is depending on the matrix itself as well as the procession method.^37–41^ These fillers also lead to improved creep resistances and strength.^6^ Changes of several properties are dependent on the dispersion of the fillers.^36^

Polymers can be used as self-lubricating solid materials. A reasonably low COF is achieved without the need of external added lubricant.^42^ Various examples for self-lubricating polymer composites are known, many of them come along with an improvement of mechanical properties, especially when synergistic effects between components are used.^31,43–48^ In tribological systems, such materials are highly reliable due to their constant lubricant concentration with no need external lubrication.^49^ An example for a self-lubricating polymer composite was introduced by Kerche *et al.*, in which liquid IL inclusions were hosted in a polyaramid-matrix.^7^ The IL was released during the friction process, which leads to a decrease in COF until a constant level is reached. Another example for self-lubrication is the combination of *in situ* generated modified multilayered graphene (MLG) and various imidazolium-based ILs as reported by Fu *et al.*^50^ This shows the synergism between graphene and ILs, based on reversible physisorption of the IL on the surface of graphene sheets which leads to an further decrease of COF in comparison to neat graphene. A self-lubricating polymer composite with synergistic effects between a carbon filler and an IL was introduced by Tang *et al.*^51^ In this material, microcapsules of poly-dopamine functionalized CNTs in a poly(urea-formaldehyde)-matrix which encapsulates lubricant oil is reported to be self-lubricating. This example also shows the effect of fillers on thermal and mechanical properties, for example the decrease in wear and decrease in tensile strength with filler content. In addition, there are self-lubricating ceramic solid materials for use at high temperatures.^45^ In general, the characteristic decrease of the initial COF on a constant level can be regarded as valid indicator for self-lubrication.

However, the processability of the combination of a solid polymer-matrix and a liquid lubricant is difficult due to the necessary non-solubility of the lubricant in the matrix. To achieve this, different methods can be applied, especially combination of a solid filler like graphene or carbon black (CB) and a lubricant which is adsorbed on the surface of the filler. Other methods are the encapsulation of a lubricant in droplets or lamella structures.^7,52–54^

The improved electrical conductivity in combination with reduced COFs, especially in self-lubricating systems, could enable a future application in electrically encumbered bearings. Typical drawbacks in such applications are breakdown voltages for bearings with low electrical conductivity which damages the bearings.^55^ Various different mechanisms are known for the formation of surface charges, depending on the interacting surfaces and lubricants.^17,21–23^ The accumulation on the surface is due to the non-conductivity of conventional lubricants, as well as the polymers themselves.^55^ The effect of generated surface charges due to different polarities on the interacting surfaces is also known as triboelectrification. The negative effect of the triboelectrification on COF and wear can be diminished by grounding the surfaces as reported by Luo *et al.*, which further highlights the importance of electrical conducting tribosystems.^17^

In this work, we use the above mentioned approach of a solid carbon-based filler (MWCNTs and CB) in combination with the long-chain alkyl-phosphonium-based IL trihexyltetradecyl-phosphonium docusate [P_66614_][DOC] in a PP matrix. The goal is to identify a PP composite, which shows self-lubricating and electrically conducting properties. The combination of the mentioned materials should combine the advantages of PP as a cheap material with the electrically conducting and self-lubricating properties of carbon-based fillers and ILs with reduced friction and wear. The polymer compounds are tribologically tested at different load collectives to investigate the mechanisms leading to the release of the lubricant (solid, liquid) and the resulting interactions of the lubricant with the sliding surface. Therefore, the composites were tribologically investigated with two geometries (ball on plate and cylinder on plate) and at different sliding frequencies to determine the influence of initial contact pressure and average sliding velocity (20 Hz = 0.04 m s^−1^ and 50 Hz = 0.1 m s^−1^) on tribological behavior. This revealed high sensitivity of self-lubricating properties on testing parameters. The composites were analyzed by scanning electron microscopy (SEM), atomic force microscopy (AFM) Raman and ATR-FTIR spectroscopy to analyze the tribological mechanisms.

## 2. Experimental

### 2.1 Preparation of composites

Carbon based (solid) fillers (CB Chezacarb AC60 (Co. Unipetrol), MWCNT NC7000 (Co. Nanocyl)) were incorporated into the polymer matrix PP (Polypropylene 575P (Co. Sabic)) using a ZSE 27HP co-rotating twin screw extruder (Co. Leistritz) with a screw diameter of 27 mm and a caliber length of 52 mm. For both carbon-based fillers, the same set of process parameters was selected, including a throughput of 12 kg h^−1^, a barrel temperature of 200 °C and a screw speed of 200 rpm. In both cases, polymer and carbon component were dosed continuously by weight in the extruder main feed and side feed, respectively. The processed compounds were cooled in a water bath and pelletized for further processing. A carbon content of 2.4 wt% for the nanotube composite and of 5 wt% for carbon black was chosen, resulting in a total amount of 4 kg for both composites.

In a second processing step, the IL ([P_66614_][DOC], Co. Iolitec) component (liquid) was incorporated into the polymer carbon composites with a Polylab QC Lab mixer system (Co. HAAKE). To achieve the desired contents of carbon an IL the sample, the pre-produced compounds were mixed with plain PP (dilution polymer) and the IL according to the ratios shown in Table 1. For the actual mixing, a sample with an overall weight of 48 g was prepared for each composite. The polymer components of the sample were pre-melted in the mixing bowl at 190 °C at a rotor speed of 50 rpm. The IL was introduced into the mixing bowl and the rotor speed was increased to 150 rpm. After 3 min of mixing, the process was stopped, the melt was removed from the mixing bowl and directly transferred to a hot press (Co. Collin) for sample production.

All used composites from polypropylene (PP), multi-walled carbon nanotubes (MWCNTs), carbon black (CB) and ionic liquid (IL) and the abbreviations

**Table:** 

PP-content/wt%	MWCNT-content/wt%	IL-content/wt%	Abbreviation
100.0	0.0	0.0	A-0 a
99.0	0.0	1.0	A-1
98.0	0.0	2.0	A-2
97.5	0.0	2.5	A-3
99.0	1.0	0.0	B-0
98.0	1.0	1.0	B-1
97.0	1.0	2.0	B-2
97.6	2.4	0.0	C-0
96.6	2.4	1.0	C-1
95.6	2.4	2.0	C-2
95.1	2.4	2.5	

a This sample was prepared by different preparation methods, whereas the sample prepared by an extruder and injection molding is denoted as C-3e and A-0e.

**Table:** 

PP-content/wt%	CB-content/wt%	IL-content/wt%	Abbreviation
99.0	1.0	0.0	D-0
98.0	1.0	1.0	D-1
97.6	2.4	0.0	E-0
95.1	2.4	2.5	E-3
95.0	5.0	0.0	F-0
90.0	5.0	5.0	F-5
85.0	5.0	10.0	F-10
80.0	5.0	15.0	F-15

Samples were produced using a template with four disk shaped cavities with dimensions of 60 mm × 1 mm. The melt was preheated to 190 °C. After closing the press, the samples were cooled at a rate of −15 °C min^−1^ until a temperature of 60 °C was reached. Samples were removed from the press and stored for at least 48 h under standard climate condition. All chemicals were used as provided by the supplier.

### 2.2 Friction and wear tests

Tribological tests were performed using a reciprocating friction and wear testing machine SRV IV (Co. Optimol Instruments Prüftechnik GmbH). A cylinder on plate geometry was used, as well as a sphere on plate geometry (Fig. S1a and b, ESI†). The average roughness *R*_a_ of the cylinder was 0.26 μm with 15 mm in diameter and 22 mm in length providing a contact length of 15 mm (standard test specimen from Optimol Instruments Prüftechnik GmbH). The balls had an average roughness of *R*_a_ = 0.26 μm and 12.7 mm diameter. Before the test, the polymeric specimen was attached on a 100Cr6 steel plate with 24 mm diameter. The tests were performed at 24 ± 1 °C, relative humidity of 30 ± 5%, reciprocating frequency of 50 Hz or 20 Hz, an initial contact pressure of 7.2 N mm^−2^ for the cylinder on plate geometry and 50.5 N mm^−2^ for the sphere on plate geometry with an applied load of 10 N. The length of stroke from each reciprocating cycle was 1 mm. Each test was carried out for 30 min. For more information on the chosen parameters, see Table S1 (ESI†). COF was evaluated by averaging the COF received from the software on the last 50 cycles of each measurement. For every specimen three measurements were carried out. Wear was evaluated for the specimens using a ball on plate geometry and was measured with a VK-9710K Color 3D-Laser scanning microscope (Co. Keyence Corporation).

For friction and wear tests with applied electrical field, the parameters were adjusted to a reciprocating frequency of 1 Hz and an applied load of 10 N. A ball on plate geometry was chosen as depicted in Fig. S1c (ESI†). A rectangular current collector loop in a distance of 1.0 ± 0.1 cm was melted on the surface of the specimen by heating. A VersaSTAT 3F Potentiostat Galvanostat (Co. Ametek Scientific Instruments) was used for applying electrical fields.

### 2.3 Characterization

All surface resistance measurements were performed with a 2400 Sourcemeter (Co. Keithley Instruments) with a 4-wire sense mode configuration in combination with a custom made 4-point measuring probe. The cylindrical probe tips were arranged in line with a diameter of 0.8 mm and a distance between the probes of 2.1 mm. A constant current was applied through the outer probe tips to the sample. The voltage drop from the outer to the inner probe tips was used for resistance calculation based on a method described by Schroder *et al.*^56^ Each measurement was performed with a constant current in the range of 1 μA to 1 mA at 2.1 V for 200 s of measurement time. The average value of 6 measurements at different locations on the sample surface is reported for each of the samples.

Attenuated total reflection Fourier transformed infrared spectroscopy (ATR-FTIR) was recorded with a 1.9 cm^−1^ spectral resolution on a 670 FT-IR spectrometer (Co. Varian Inc. (now: Agilent Technologies)). The assignment of measured vibrations were supported by DFT-based calculations on a D3(BJ)-BP86-def2-SVP level of theory.^57–61^ Deviations to the measured spectra were described by Benavides-Garcia and Monroe.^62^ All calculations were carried out using the ORCA computational chemistry program.^63,64^ Raman spectra were recorded on an inVia confocal (Co. Renishaw) with an excitation wavelength of 532 nm, 3 times for each specimen with 20 s exposure time. Hardness testing was performed using a Fischerscope H100C XYp Nanoindenter (Co. Helmut Fischer GmbH) using a Vickers diamond indenter. After contact with the surface, the indenter was approached into specimens at a constant rate of 300.00 mN/60 s until 150 mN of force was reached and withdrawn from the surface at the same rate as loading. At least 12 indentations were performed for each specimen and the average value was reported. Surface roughness measurements and optical imaging were performed using a VK-9700 Color 3D-Laser scanning microscope (Co. Keyence Corporation). For each sample, at least five randomly selected areas of the surface were measured and the surface roughness *R*_a_ and surface depth *R*_z_ were determined. High-resolution images of the composite material were taken using a scanning electron microscope (SEM, S-3400N, Co. Hitatchi Science Systems, Ltd) and spectral maps for sulfur and phosphorus were prepared using energy dispersive X-ray spectroscopy (EDX). The samples were fractured after storage in liquid nitrogen for at least 3 h and the exposed surface was coated with a thin platinum layer using a high vacuum platinum sputter at low voltage (brittle fractures). High-resolution transmission electron microscopy (FEI, Talos 120C, Co. Thermo Fisher Scientifics) images were taken of selected polymer compounds. Therefore, very thin lamellae were sectioned with a diatome diamond knife (Cryo-Mikrotomy, Co. Reichert-Jung Ultracut E and RMC CR-X Cryoattachment) at a temperature of −120 °C. The freshly microtomed sample surfaces were subsequently measured by AFM (MultiMode 8, Co. Brucker). The ultrathin sections (about 60 nm) were collected and used for TEM measurements. By evaluating the distribution of the added liquid and solid lubricants in the bulk material, the tribological mechanisms leading to self-lubrication will be analyzed.

## 3. Results

### 3.1 Hardness and electrical surface resistance

**Fig. 1:**
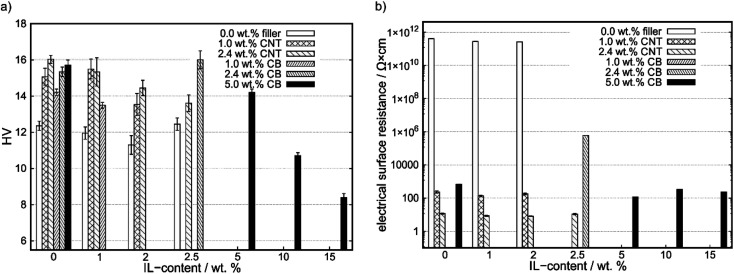
(a) Vickers hardness (HV) as a function of IL-content for different carbon contents. (b) Electrical surface resistance as a function of IL-content.

The results of the hardness measurements are shown in Fig. 1a. With increasing filler-content the hardness increases, as shown in the row A-0, B-0, C-0, as well as A-0, D-0, E-0, F-0. However, the hardness of CNT-containing compounds is higher than the one of CB-containing compounds at same filler-contents. By adding IL to the compounds, the CB-containing compounds do not strictly show lower hardness values in comparison to their CNT counterparts, as shown for E-3 and C-3. With increasing IL-content, the Vickers hardness (HV) decreases for constant carbon contents (see: C0 to C3, F0 to F15) except for A-3 and E-3. In Table S2† the exact values for Vickers hardness and electrical surface resistance are shown, as well as COF values (see 3.2 Tribological characterization).

In Fig. 1b electrical surface resistances are shown. Compounds with no reported surface resistances could not be measured with the four-point method and show higher resistances than 1 × 10^6^ Ω cm.

The electrical surface resistance of compounds of PP does not change significantly with IL-content. For increasing filler-content the surface resistance significantly decreases. By comparing the compounds with 0.0, 1.0 and 2.5 wt% IL-content for different fillers the CNT-containing compounds show a lower electrical surface resistance and, therefore, an increase in surface conductivity at the same filler content than CB-containing compounds.

### 3.2 Tribological characterization

**Fig. 2:**
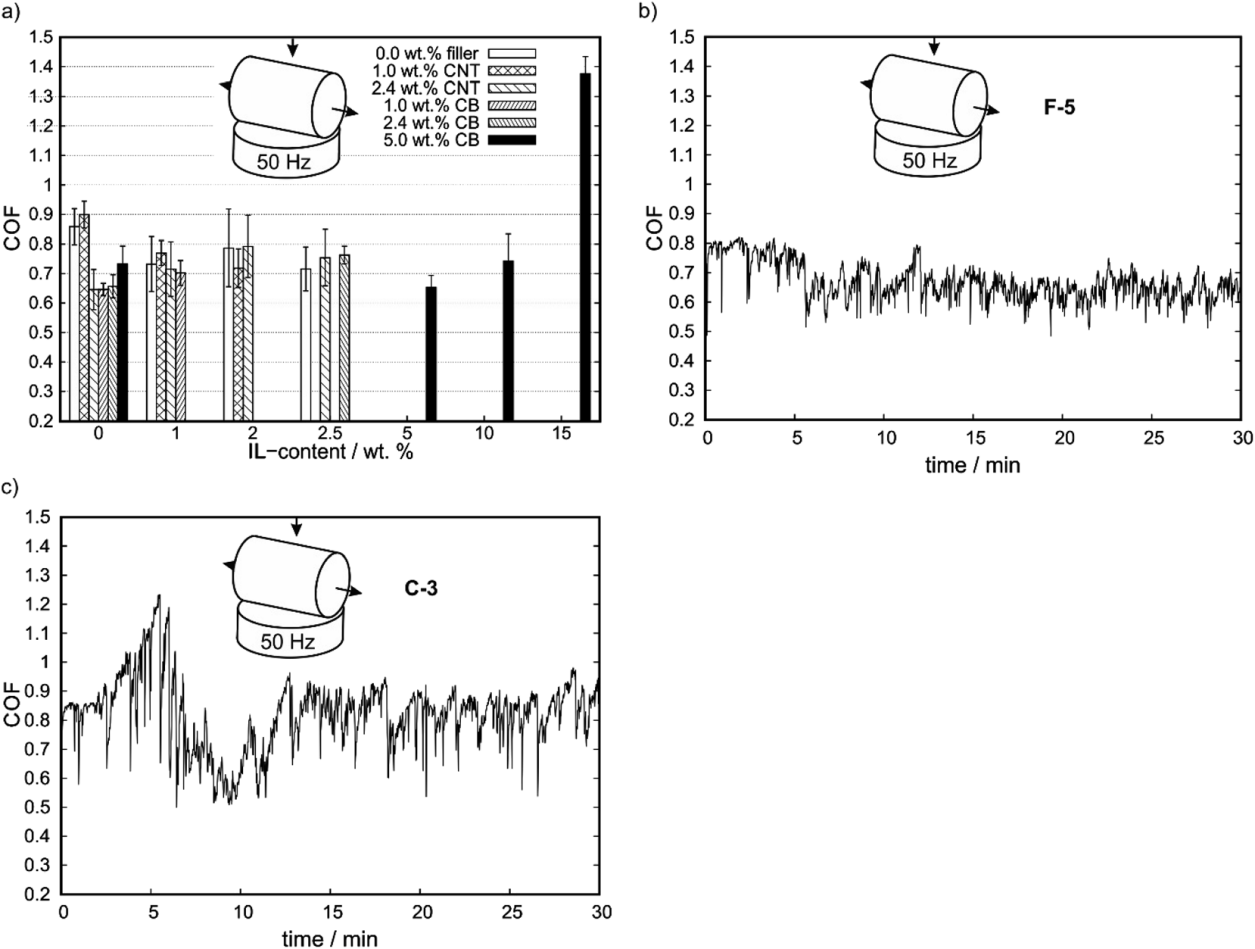
(a) Averaged COF over the last 50 s of measurement of the compounds for cylinder on plate geometry at 50 Hz reciprocating frequency after 30 minutes of testing. (b) An example measurement at 50 Hz of reciprocating frequency for the F-5 compound over the full measurement time and (c) for the C-3 compound with a cylinder on plate geometry.

In the first step, the friction behavior was investigated with a cylinder on disc geometry to demonstrate the possible self-lubrication properties at relatively low initial pressures. An estimation of the contact stress (Hertzian stress) at 10 N normal force results in an initial contact pressure of approx. 6.7 MPa with PP (Young's modulus: 1300 MPa, Poisson's ratio: 0.42, datasheet PP). The coefficients of friction (COF) of the different compounds for a cylinder on plate geometry at 50 Hz reciprocating frequency are shown in Fig. 2a. With a cylinder on plate geometry, IL-free compounds showed a decrease in COF in comparison to PP with a few exceptions. For increasing CB-contents (D-0, E-0, F-0), COF increases. By addition of IL to B-0, COF decreases with IL content. The CNT-based C-0 shows an initial increase in COF until a maximum is reached at 2.0 wt% IL content, before the COF decreases again with increasing IL content. For CB-based compounds, a similar behavior is observed with a minimum in COF for F-5. For higher IL-contents, the COF increases again. The decrease in COF implies the release of IL into the wear trace, whereas the low wear at these low contact pressures allows for self-lubrication, as discussed in the following. At high IL contents the mechanical weakening of the matrix might cause increasing COFs again. In these measurements, the error bar is an indicator for the stability of the COF. For reference, in Fig. 2b and c, the full measurements for a CB- and a CNT-containing compound with same IL- and filler-content are shown. The COF of CB-containing samples showed generally smaller scattering in comparison to the CNT-compounds and no abrupt COF changes. In comparison to that, C-3 (Fig. 2c) with 2.4 wt% CNT- and 2.5 wt% IL-content shows a higher error bar on the averaged COF values than F-5 (Fig. 2b) which is in line with the scattering of each COF measurement. Furthermore, for the cylinder on plate geometry and CNT-containing compounds the COF started at a distinct level and stayed constant for the test duration with a strong scattering and several COF changes around the average value. For CB-containing compounds a stable COF was reached after a period and kept constant afterwards. This is the typical behavior of self-lubricating materials (see Section 4.1). Furthermore, a reduction of up to 25% in COF in comparison to A-0 was achieved for C-3. Further reduction in COF was observed for samples prepared by injection molding. This also influences the (surface) hardness, roughness and electrical surface conductivity. For more information, see Fig. S2 and S3 (ESI†).

Since self-lubrication could be demonstrated with the cylinder-on-disc geometry, the next step was to investigate the influence of contact pressure and velocity on tribological behavior. By changing the counter body to a ball, the initial contact pressure increases to approx. 49 MPa with PP. In order to investigate the limits of self-lubrication by reducing the Stribeck parameter, in addition the sliding speed was decreased. This gives a wide variation of several orders of magnitude of the quotient of average velocity *v* and contact pressure *p*, which results in the Stribeck parameter by multiplication with the lubricants viscosity. This parameter characterizes the friction regime in conventional tribological systems.

**Fig. 3:**
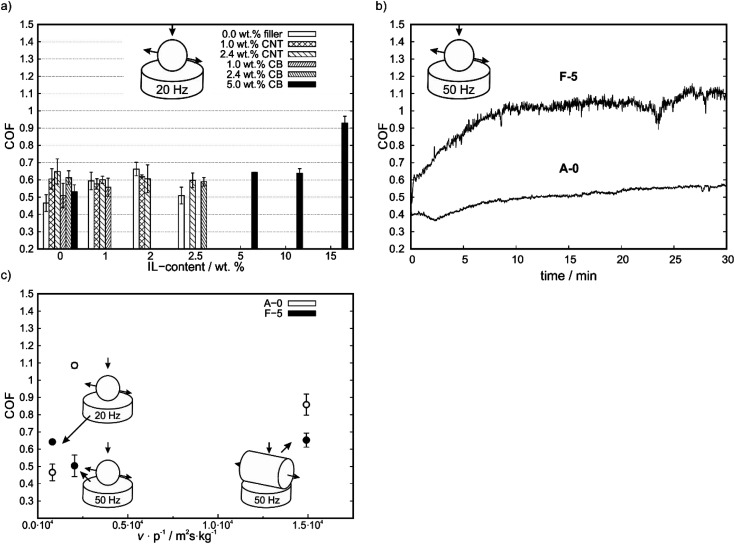
(a) Averaged COF over the last 50 s of measurement of the compounds for ball on plate geometry at 20 Hz reciprocating frequency after 30 minutes of testing. (b) Course of COF over time for F-5 (higher COF) and A-0 (lower COF) at 50 Hz of reciprocating frequency with a ball on plate geometry. (c) Effect of frequency and contact geometry (described by the quotient of sliding velocity *v* and initial contact pressure *p*) on mean COF after 30 minutes of testing for each geometry.

As shown in Fig. 3a, at reduced reciprocating frequencies and increased contact pressure the addition of any kind of filler increases the COF. Furthermore, the addition of IL to a either filler containing or non-containing matrix did not lead to a significant reduction of COF in comparison to neat PP. Nevertheless, in comparison to the IL-free matrix a reduction in COF at high IL contents were observed for A-3, C-3 and E-3. The high contact pressure in this geometry might cause IL displacement out of the wear trace and strong mechanical deforming of the matrix, thus generating high wear.

To determine whether the change in geometry or the change in reciprocating frequency is responsible for major changes in COF, additional tests with ball on plate geometry at 50 Hz were conducted (Fig. 3b). In these experimental conditions, the Stribeck parameter is reduced by 7 times compared to the geometry with cylinder at 50 Hz. For these tests PP (A-0, reference material) and F-5 (lowest COF among all IL-containing compounds) were selected. Under those conditions, a COF of 0.50 ± 0.06 for neat PP and 1.09 ± 0.02 for F-5 was measured. With the ball, no run-in associated with a decrease in COF is observed as with the cylinder, thus no self-lubrication is realized which shows the parameter dependence of self-lubrication (Fig. 3b).

To determine the frequency and pressure dependence of the COF, a proportionality factor corresponding to the Stribeck parameter can be determined from the quotient of the average sliding velocity *v* and the initial contact pressure *p* (cylinder-50 Hz 1.49 × 10^4^ m^2^s kg^−1^, ball-50 Hz 2.04 × 10^3^ m^2^s kg^−1^ and ball-20 Hz 8.16 × 10^2^ m^2^s kg^−1^), as shown in Fig. 3c. This shows a U-shaped course for the self-lubricating F-5 compound with a minimum at *v p*^−1^ = 2.04 × 10^3^ m^2^s kg^−1^. For the pure matrix A-0, this ratio results in a maximum. The COF increases for higher *v p*^−1^ values and no indications of self-lubricating were obtained at any *v p*^−1^ parameter.

In general, effects on COF by addition of any additive are more pronounced for the cylinder on plate geometry at a higher reciprocating frequency whereas for lower frequencies with a ball on plate geometry only small changes are measured. This indicates a high dependency on testing conditions for self-lubricating composites.

Furthermore, tests with applied electrical potential have been conducted in order to simulate accumulating surface charges. The results of the surface analysis are shown in Section 3.5.

### 3.3 Wear behavior

**Fig. 4:**
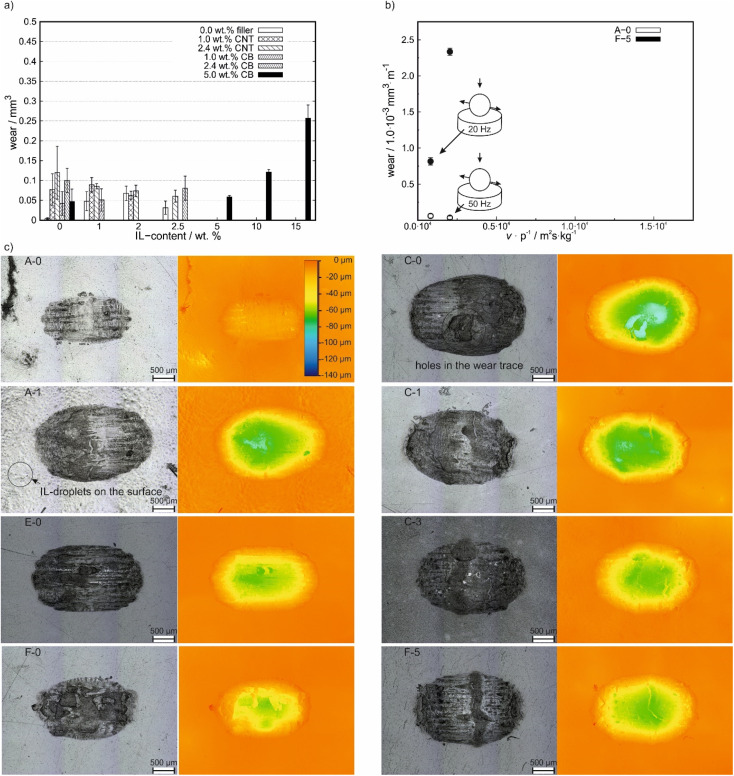
(a) Wear analysis after 30 minutes of testing at 20 Hz of reciprocating frequency with a ball on plate geometry. (b) Comparison of wear volumes at different average sliding velocities. (c) Corresponding images of the wear trace to Fig. 4a of A-0, A-1, E-0, C-0, C-1, C-3, F-0 and F-5 compound after testing with a ball on plate geometry at 20 Hz of reciprocating frequency and the corresponding height maps.

In Fig. 4a the wear volumes after friction tests at 20 Hz using ball on plate geometry are shown. Due to a lack of accuracy for long wear tracks, the wear volume could only be measured for small wear tracks. Due to this, the determination of the wear volume was only possible for the tests with the ball on plate geometry. For filler free compounds, the wear volume increases for small amounts of added IL before it decreases again with a maximum for 2.0 wt% IL content. For all other IL-free compounds, the wear volume is higher than for neat PP and decreases with the IL content slightly before it increases again at high IL-contents, especially above 5.0 wt%. For none of the compounds a reduction of wear volume in comparison to neat PP was achieved. Nevertheless, with increasing carbon-filler content the wear volume decreased, whereas the CNT-based filler showed a stronger reduction in wear at similar amounts added filler to the composite in comparison to the CB-containing composites except for IL-free compounds.

The frequency dependence of the wear volume on the polymer plate is shown in Fig. 4b. To take the different sliding distances (due to different frequencies and similar testing durations) into account, the wear volume was normalized on the sliding distance. Under both testing conditions, lower wear values were obtained for A-0. Furthermore, a decrease in wear volume is observed for A-0 for increasing *v p*^−1^ values, whereas a significant increase is observed for the CB containing F-5.

In Fig. 4c, laser images and the corresponding height maps (with which the wear volume was measured) of several compounds are shown (all other compounds are shown in Fig. S4 (ESI†)). Compared to the pure PP (A-0), all samples showed an increase in wear volume, which was accompanied by an increase in the maximum depth of the wear track. However, E-0 containing 2.4 wt% CB showed only a slightly higher wear volume compared to A-0, as shown in Fig. 4a. For the 2.4 wt% CNT-containing C-0, an increase in wear volume compared to A-0 and several holes in the wear track were observed. These holes decrease in depth with the addition of IL as seen for C-1 and C-3. By the addition of IL to the neat PP matrix (as seen for A-1), the depth of the wear trace increases. Furthermore, small droplets/bubbles are observed on the surface.

### 3.4 Surface analysis

**Fig. 5:**
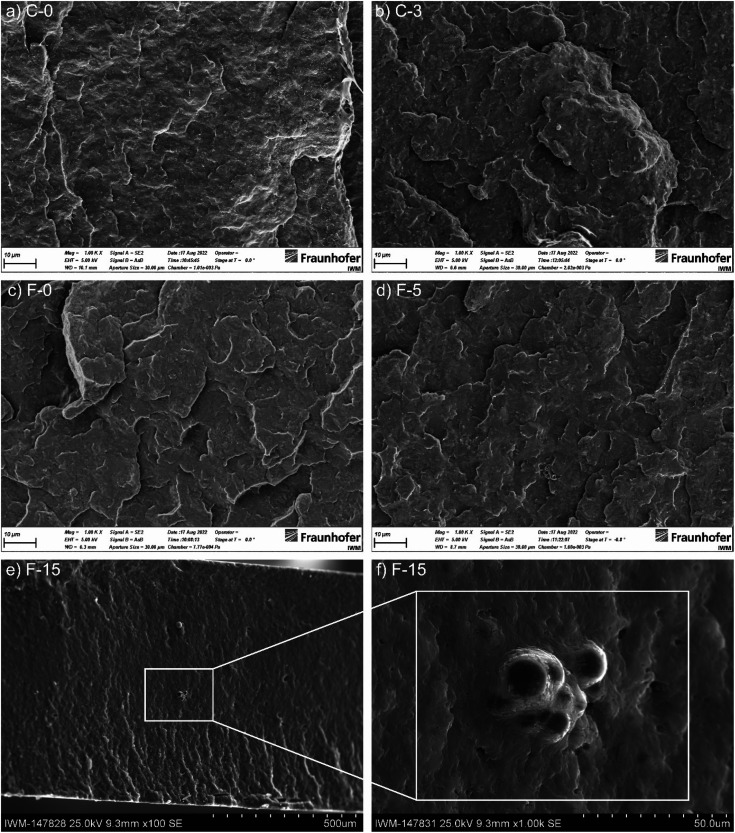
SEM micrographs of brittle fractures of (a) C0, (b) C3, (c) F0, (d) F5 and (f) F15 at a magnification of 1000 at 25.0 kV after storage in liquid nitrogen. (e) SEM micrograph of brittle fracture of the F15 compound at a magnification of 100.

Fig. 5 shows scanning electron micrographs (SEM) of the brittle fracture of CNT-containing compounds C-0 and C-3 and CB-containing compounds F-0, F-5, and F-15. C-0 and F-0 show similar homogeneous morphologies, with no significant changes upon IL addition (C-3 and F-5). In contrast to that, for high IL-contents the formation of droplets is observed in the bulk (see Fig. 5f). Furthermore, for this sample smoother crack-edges were observed. High-resolution transmission electron microscopy (TEM) images are shown for B-0, B-1 and B-2 in Fig. S5 (ESI†). TEM thin sections were generated by cryo-microtomy. Water was used to collect the sections on the TEM grids (standard sample preparation for polymers and TEM studies). This presumably removed the IL from the sections and therefore could not be detected. In contrast, the CNT is clearly visible.

**Fig. 6:**
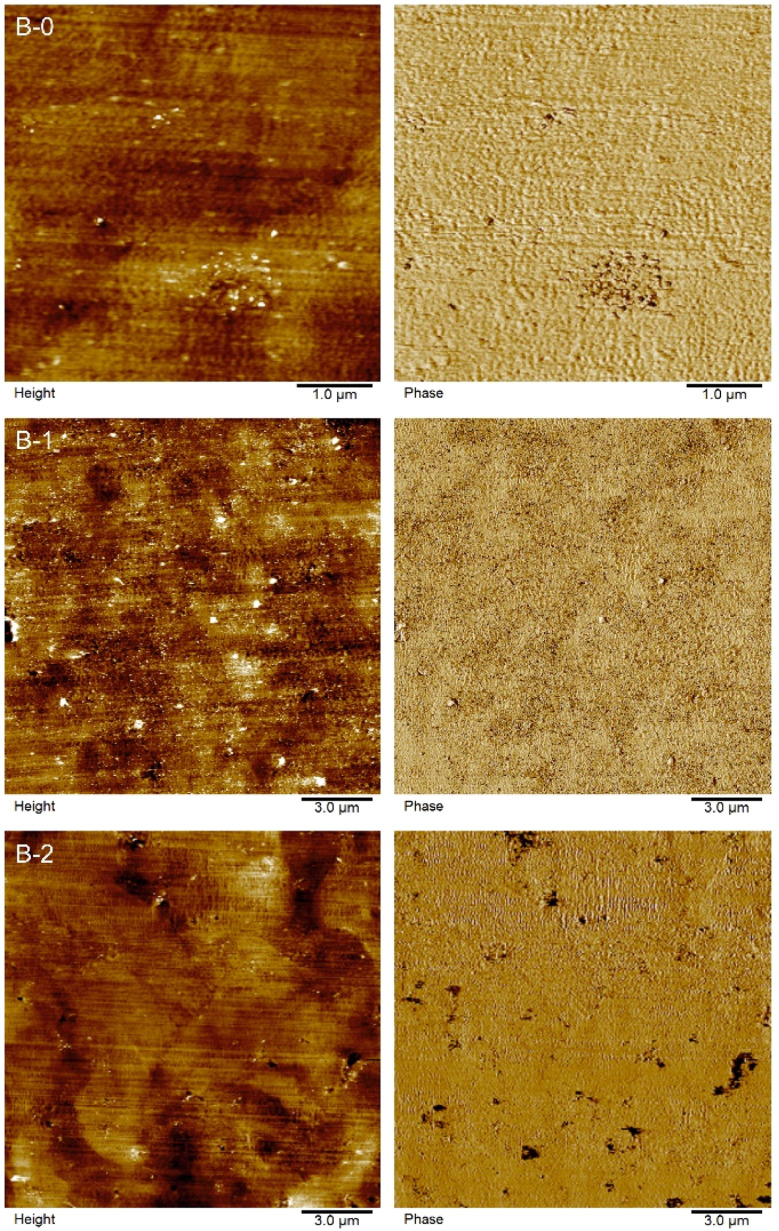
AFM-height and corresponding phase images of the B-0, B-1 and B-2 compounds.

Atomic force micrographs (AFM) are shown for B-0, B-1 and B-2 in Fig. 6. Phase-images show small, agglomerated areas for B-0, which are highly likely related to CNT agglomerates. The addition of IL leads to a more homogeneous distribution of these areas (B-1). Higher IL contents lead to phase segregated areas of soft areas, implying the formation of IL-rich areas (dark areas) rather than a homogeneous distribution. The IL seems to be dissolved completely into the polymer.

**Fig. 7:**
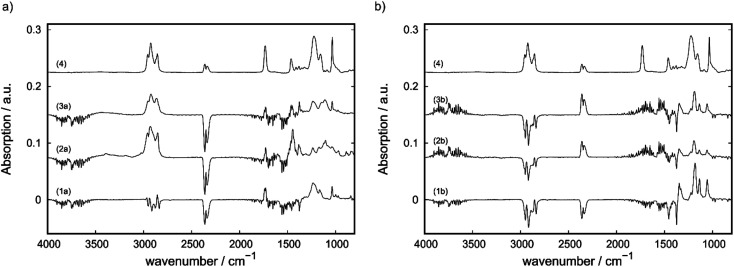
(a) ATR-FTIR spectra of CNT- (C-3) and (b) a CB-containing (E-3) compound before (2a, 2b) and after a wear test (3a, 3b), as well as for pure IL (4) and neat PP with 2.0 wt% IL-content (1a) and 2.5 wt% IL-content (1b).

Differential-ATR-FTIR spectroscopy and Raman spectroscopy were applied to characterize the interaction of IL with the matrix as well as to characterize changes on the sample surface within the tribological tests. The IR-spectra as obtained after subtracting the spectra of IL-free matrix (A-0 for filler free compounds and C-0 or E-0 for CNT-containing and CB-containing compounds) are shown in Fig. 7. The filler-free A-2 (1a) and A-3 (1b) show similar spectra to the free IL (4) after subtracting the spectra of pristine PP. For C-3 ((4a), (2a) and (3a)) a shift of the band at 1240 cm^−1^ towards lower wavenumbers is observed before the wear test (spectra 2a). This shift gets partially reversed during the wear test as seen in spectra 3a where the shoulder at ∼1145 cm^−1^ is still observed, but with a lower intensity in comparison to the band assigned to the free IL at 1240 cm^−1^. With the support of DFT-calculations the associated mode could be identified as a phosphorus-alkyl deformation mode (for more information see Fig. S6 (ESI†)). In comparison to the CNT-containing C-3, the spectra for CB-containing E-3 ((4b), (2b) and (4b)) do not show changes after the tribological test. In comparison to filler-free A-3 no differences could be observed.

**Fig. 8:**
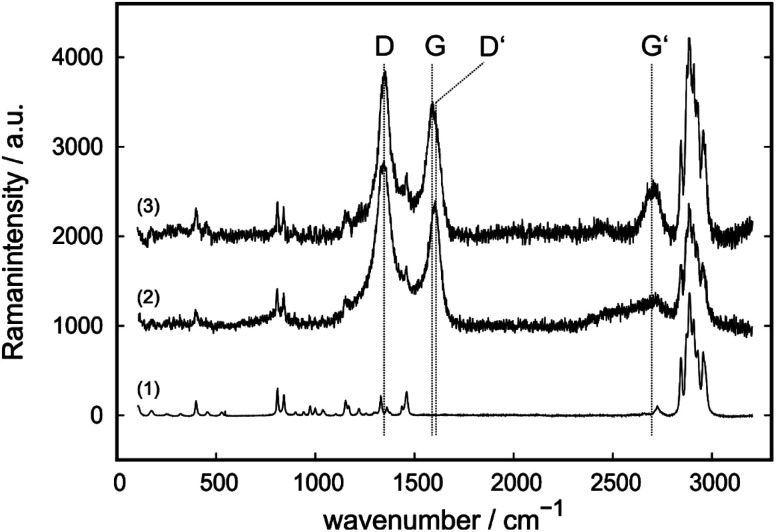
Example Raman spectra as received from the compound containing no carbon filler (1), CB-containing E-1 compound (2) and CNT-containing C-3 compound (3) after the wear test.

To identify changes of the carbon filler component within different tribological test conditions, Raman spectroscopy was employed as shown for one filler free, one CB-containing and one CNT-containing compound (Fig. 8). The typical frequencies for PP were measured in all spectra.^65^ As discussed in the introduction, the typical features of carbons can be observed within the spectra (Fig. 8-(2) and (3)). However, the D′ band at ∼1615 cm^−1^ is only resolved for CNT-containing specimens.

The intensity ratios *R* are given in Table 2 for C-0, C-3, E-0 and E-3 as well as after tribological tests with and without applied potential. This gives insights, whether the degradation mechanisms in the material change with the application of an external potential. Due to this, information about possible degradation reactions as a result of breakdown voltages is obtained. Due to the fact, that the exact position of those bands and their intensities are influenced by laser excitation energy and wavenumber, it is crucial to compare only spectra measured with the same settings. For the IL-free compounds C-0 and E-0, and C-3 after tribological testing with externally applied potential, the *R* ratio decreases during the friction test. For all IL-containing compounds under normal testing conditions the ratio increases, as well as for E-3 under tribological testing conditions with applied potential.

**Table:** Intensity ratios for several specimens before and after a wear test under different testing conditions

Compound and conditions^a^	Position on the sample	*R* b
C-0 (2.4 wt% CNT)	Pristine	1.65
	Wear trace	1.51
C-3 (2.4 wt% CNT and 2.5 wt% IL)	Pristine	1.27
	Wear trace	1.39
C-3 (2.4 wt% CNT and 2.5 wt% IL) after wear test with applied potential	Pristine	1.47
	Wear trace	1.46
E-0 (2.4 wt% CB)	Pristine	1.90
	Wear trace	1.80
E-3 (2.4 wt% CB and 2.5 wt% IL)	Pristine	1.85
	Wear trace	1.95
E-3 (2.4 wt% CB and 2.5 wt% IL) after wear test with applied potential	Pristine	1.87
	Wear trace	1.91

a If not stated otherwise the tribological testing was performed without applied potential with a ball on plate geometry.

b All fits spectra were fitted until a coefficient of determination *R*^2^ > 0.99 was reached.

## 4. Discussion

### 4.1 Mechanical properties and electrical surface resistance

As reported in the literature, adding fillers like carbon black and carbon nanotubes to a polymeric matrix leads to an increase of hardness and other mechanical properties.^10,33^ This is due to the high elastic modulus of those fillers and is in line with our findings on increase in hardness by adding a carbon filler to the matrix, which are shown in Fig. 1a. However, the increase in hardness is higher by adding MWCNT than for CB-based fillers, which may be due to the stiffer backbone of MWCNTs in comparison to amorphous CB. By adding IL to a matrix, the hardness decreases with an exception for A-3. For this compound a similar hardness as for the neat PP can be reported. This may be due to difference in bulk and surface concentration of additives and needs further investigations. Whereas the literature reported synergistic effects between carbon fillers and ILs on mechanical properties, this is not the case in the system which was used in this work.^29,30,33^ Here, we cannot report an increase in hardness for any given composition containing IL in comparison to the IL free matrix with the filler. Instead, the hardness decreases with increasing IL content. This implies that a certain amount of IL is dissolved within the matrix, or the IL decreases the stabilizing interaction of the filler with the matrix, either of which destabilizes the mechanical properties of the compound thus explaining increasing COF values.

The electrical surface resistance decreases by adding carbon fillers to the PP matrix as shown in Fig. 1b, whereas for a given composition the MWCNT containing compounds show lower surface resistances in comparison to their CB counterpart due to their tube-like structure. This has been reported various times, however, for small carbon contents and relatively high IL contents also the IL influences the conductivity.^37–41^ This can be seen for E-3 with an electrical surface resistance of 6.1 × 10^5^ Ω cm in comparison with E-0 which shows an electrical surface resistance outside of the measurement window and, therefore, a value exceeding 1.0 × 10^6^ Ω cm. Furthermore, the same behavior is observed for F-0, F-5, F-10 and F-15 where surface resistances differ from each other. Notably, this dependency of electrical surface conductivity on IL-content is only observed for CB containing compounds and not for CNT containing ones. This may be due interactions between the filler and our chosen IL like chemisorption or adsorption of weather the cation or the anion on MWCNT-surfaces. Therefore, the local structure of adsorbed IL as well as the binding situation might change.

### 4.2 Tribological characterization

Self-lubricating polymer compounds could be identified. Tribological characterization of the compounds revealed a fundamental difference for the polymeric compound systems depending on the selected contact geometry and reciprocating motion frequency, as shown in Fig. 3c. This is in line with earlier findings for expanded graphite – carbon black systems in acrylonitrile-butadiene (NBR) rubbers.^36^ For simplicity, the geometry ball on plate will be referred to as point contact and the geometry cylinder on plate as line contact in the following. For a line contact, the addition of IL to the filler free matrix results a reduction in COF, which implies a possible usage of ILs for solid-state self-lubricating polymer composites. This was shown by comparing the neat matrix A-0 with the COFs of A-1, A-2 and A-3 which are comparable to each other. The reduction in COF by adding IL to the composite implies the release of free lubricant in the wear trace. The addition of IL to a filler containing compound leads to an increase in COF for small amount of IL and to a decrease in COF for higher amounts of added IL. This is not observed for all tested materials. However, it is assumed that this trend depends on the ratio of carbon to IL and therefore the amount of available IL. For small carbon to IL ratios no IL can be released during the wear tests due to strong adsorption or chemisorption on the carbon surface and especially on defects. This is inline with AFM results, which show a phase segregation only for high IL to CNT ratios (see Fig. 6). Therefore, the loss of mechanical stability (as measured by the hardness reduction of those compounds, see Fig. 1) due to IL in the compound explains the increase of COF until a certain limit. After saturation of the adsorption centers, the enthalpy for releasing the IL from an adsorbed state decreases which leads to free IL in the wear trace and therefore a reduction of COF. For high IL contents the COF increases again due to mechanical weakening of the matrix. This is in line with the bubbles observed in brittle fracture SEM micrographs for F-15 (see Fig. 5e and f), as well as phase segregation observed in AFM phase-images (Fig. 6). Therefore, a minimum in COF was reached for F-5, D-0 and C-0 which corresponds to a reduction of up to 25% in comparison to A-0 to a total value of 0.65 ± 0.04. As highlighted in Fig. S7 (ESI†), it is possible to further reduce the COF by different preparation methods.

As shown in Fig. 2 and 3 not only the IL influences the tribological behavior but also the carbon source. For a line contact at high reciprocating frequencies, the IL-free compounds showed a decrease in COF in comparison to neat PP for any amount of added filler. This can be explained with the increasing hardness and improved mechanical properties of those compounds. Another factor may be the reported lubricating properties of graphene, carbon nanotubes and carbon black.^29,31,35,36^ Whereas for all CB containing compounds expect F-10 and F-15 the COF decreases during the measurement, the COF stayed constant for CNT containing compounds. The behavior of F-5 with decreasing COF over time is therefore typical for self-lubricating systems as shown in Fig. 2b.^7,42,44^ This implies that the tribologically released IL during the wear test reduces the COF. This is also in line with the mentioned results from filler-free compounds and their reduction in COF upon IL addition.

In Fig. 4, images and height maps of wear traces after testing with a ball on plate geometry are shown. The formation of IL-droplets or bubbles on the surface of A-1 shows, the (for self-lubrication desired) non-solubility of the IL in the PP-matrix. This changes with the addition of carbon fillers, which is in line with the lack of bubbles in SEM micrographs for C-3 and F-5 (Fig. 5b and d). However, the wear volume increases with the addition of any additive. For IL-free compounds, CB-samples show a lower wear volume than the CNT-samples, which also show holes in the wear trace. These holes explain the high wear on CNT samples. The formation of these holes is not observed for IL containing samples, which leads to a decrease in wear and implies a less hard and smoother surface. The disappearance of the holes is connected to the realized lubrication mechanism and shall be described in the following section. The smoothing of the COF evolution with time of IL and CNT-containing samples in comparison to CNT-containing, IL-free samples is also in line with this.

### 4.3 Influence of testing parameters and analysis

For tests performed with a point contact and low frequencies no reduction in COF was observed, unlike for tests performed with a line contact at high frequencies. It should be highlighted, that due to the relative softness of PP in comparison to the steel ball, plastic deformation might occur which influences the COF. At high contact pressures, the IL content does not affect the COF significantly, which means, that too less lubricant is available to reach the regime of hydrodynamic- or elastohydrodynamic lubrication and form a stable tribofilm and instead solid–solid interaction takes place.^3^ As already highlighted, for pure polymer–carbon composites such as CB and graphite in NBR rubbers a dependency on load and sliding velocity is already known.^44^ Fan *et al.* observed a decrease of COF at higher loads and higher sliding velocities.^22^ This is not in line with the results of this work. Herein, we observed a reduction in COF by 41% when changing from a line contact to a point contact for the neat PP-matrix (Fig. 2 and 3). The change from the line to point contact equals an increase in initial contact pressure by the factor of ∼7.0. For 50 Hz of reciprocating frequency with a point contact a COF of 0.50 ± 0.06 was achieved for neat PP, under similar conditions with 20 Hz reciprocating frequency a COF of 0.47 ± 0.05 was observed. The change in reciprocating frequency equals a change in sliding velocity by a factor of ∼4.0^−1^. An increase of 69% in COF for the CB and IL-containing compound F-5 by changing the geometry from a line contact to a point contact was measured. The change in COF exceeds the previous findings in the literature and are, therefore, most likely due to a change in the mechanism. It is assumed, that under those conditions the thickness of the IL film is not high enough to separate the surfaces, which means that there is no liquid lubrication. Instead, solid–solid lubrication takes place. This is in line with the typical behavior of liquid lubricants as described by the Stribeck curve.^3^ However, the Stribeck curve is only valid for liquid systems. Due to this, for solid self-lubricating systems as the one used in this work, not only the typical parameters which influence the Stribeck curve must be considered but also the amount of lubricant released from the matrix. Therefore, the accessible COF-window of the Stribeck curve as given by a lubricant and a pairing of two interacting surfaces gets reduced, or in other words, no stable tribofilm can be formed outside of this window due to the lack of lubricant. The fact that those systems are in line with the predictions of the changes in COF by the Stribeck curve by changing a certain parameter also shows that even small amounts of IL in the matrix lead to a high enough amount of free IL in the wear trace to form a stable tribofilm. It can be concluded that for a point contact with a reciprocating frequency of 20 Hz only boundary or mixed lubrication can be observed. By changing the reciprocating frequency from 20 Hz to 50 Hz, the COF changes from 0.643 ± 0.01 to 1.09 ± 0.02. This is contradictory to the typical behavior of liquid lubricants, whereas the COF should further decrease or be stable if the parameters lead to elastohydrodynamic or hydrodynamic lubrication.^3^ This depicts the limited COF-window of the Stribeck curve of liquid lubricants, which is only valid as long as sufficient amount of lubricant is available to form a stable tribofilm. The stability of this tribofilm is highly parameter dependent. For high contact pressures and low frequencies more lubricant is required to form a stable tribofilm or to realize the hydrodynamic lubrication regime.

Not only the IL influences the formation of the tribofilm, but also the carbon filler itself which dominates the behavior in solid-based lubrication regimes. Due to this, a precise prediction in the behavior of polymeric, IL and carbon filler containing systems is not possible so far. However, this work gives a guideline for the behavior of self-lubricating, solid-state materials with embedded liquid lubricant.

An explanation for the different behavior in terms of self-lubricating properties (Fig. 2) of MWCNT- and CB-based, IL containing compounds can be drawn from the differential ATR-FTIR spectra. As mentioned above, CB-containing compounds show self-lubricating behavior. This suggests the release of free IL during the wear test into the trace. This is in line with the ATR-FTIR results as shown in Fig. 7, whereas the bands assigned to the free IL can be observed in the wear trace after the wear test, as well as before the wear test. Therefore, this interaction can be described by a weak adsorption of IL on the surface of CB-domains within the sample, which does not change the electronical properties of the IL. This changes for the MWCNT-containing C-3 compound. For this sample, a shift of the band at 1240 cm^−1^ which can be assigned to phosphorous-alkyl deformation mode to lower wavelengths is measured. This implies a strong interaction between the phosphorous based cation and the CNTs within the sample, which might be based on strong adsorption or chemisorption of the IL. After the wear test, the intensity of this band at 1145 cm^−1^ is reduced and the band assigned to the free IL can be partially observed again. This indicates the partially release of IL into the wear trace. However, this seems to be limited as seen on the COF profile. The amount of released IL is not high enough to form a stable liquid tribofilm, which is necessary to reach hydrodynamic lubrication regime. This supports the suggestion mentioned above that CNTs do not show synergism in terms of tribological and electrical properties with the chosen IL. Therefore, it is crucial to examine the interactions between ILs and fillers for all applications inspired by this work. We conclude that the amount of IL released during wear strongly depends on the interaction with the filler.

(MW)CNTs and other carbon allotopes can also be characterized *via* infrared spectroscopy (IR) and Raman spectroscopy which give insights on structural changes which is therefore a useful tool.^66,67^ However, the Raman spectra of different carbons differ as discussed in the literature.^66–69^ For all allotropes of carbon, the G-band (∼1580 cm^−1^) and the G′- or 2D-band (∼2700 cm^−1^) can be observed, which corresponds to well-ordered domains.^68^ The D′-band at ∼1615 cm^−1^ is from some authors assigned to ion intercalation.^67^ Especially for carbons with low ordering, the D′ and D-band at ∼1340 cm^−1^ can be observed in higher intensities due to the defective origin of this band.^68^ Therefore, the presence of the D-band implicates structural defects whereas the G-band replicates ordered surroundings of an absorbed phonon. Due to this, the intensity ratio *R* as described in formula (1) respectively (2) if the D′-band is resolved is a valid tool for characterization of structural defects within the carbon allotropes. However, Raman absorption bands of carbon allotopes are in the same range as various components, for example polypropylene (PP).^65^

1 *R* = *I*_D_/*I*_G_

2
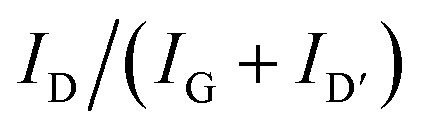


The *R* ratios of C-0, C-3, E-0 and E-3 compounds were compared before and after the tribological test, as well as after a wear test with applied potential. This was done to evaluate a possible usage as solid tribosystems with switchable COFs and to simulate accumulating charges on the surface. For all non-IL containing materials a decrease in 
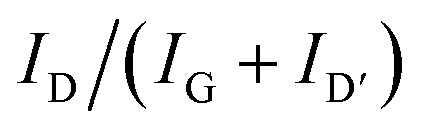
 was observed during the wear test, which indicates a loss of defects. This means that non-graphitic or non-ordered areas of the carbon seem to react faster than ordered areas during the wear process, which changes for IL containing systems. For those, an increase in the 
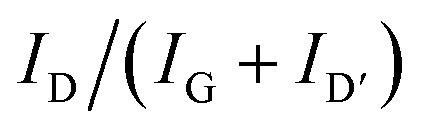
 ratio can be observed which highlights the synergism between IL and carbon. The increase is attributed to an increase of defects relative to ordered areas. The IL is interacting with the carbon filler *via* adsorption or chemisorption. This is most likely mainly due to surface interactions, since little is incorporated into the bulk of the filler, while the surface represents defects. An increase of defects during the wear test might be related to a protective layer of IL on the defective surface which explains an increase of the 
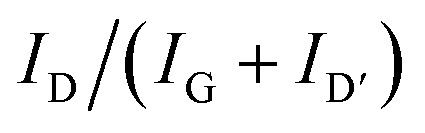
 ratio. However, the ratio increases slightly with applied electrical field for the CB-containing E-3 and decreases slightly for C-3. This highlights the difference in the behavior of CB and MWCNT. It is assumed, that due to the orientation of IL in an external electrical field, the protective layer on the surface cannot be as stable as it was before which leads to an inversion of the behavior. This seems to have a higher impact on strongly bound IL in MWCNTs than it has in CB.

**Fig. 9:**
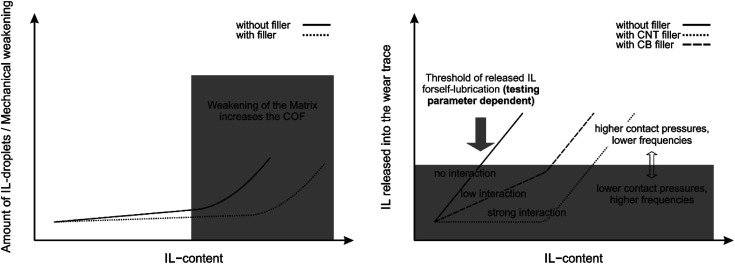
Schematic illustration of effects of filler, testing parameters and IL content on mechanical weakening/formation of IL-droplets in the matrix and available IL in the wear trace.

The summary of the above-mentioned results and their interpretation are shown in Fig. 9. Due to this, the knowledge about interactions between lubricants and fillers is key to increase the available amount of lubricant in the wear track without resulting in mechanical weakening of the matrix. Given the limited amount of IL present in the wear track, boundary and mixed friction rather than hydrodynamics is achieved for several tested compositions, which is the reason for the high influence of the testing parameters on the COF. In other words, no stable tribofilm is formed due to a lack of lubricant under these parameters. This highlights the importance of the testing parameters and opens the way for possible controllability of COF of those systems for which a liquid lubrication regime is needed. Nevertheless, a reduction in COF of up to 25% in comparison to pristine PP was achieved for the CB containing compound F-5 with a cylinder on plate geometry and 50 Hz of reciprocating frequency. It was also shown that the COF can be further reduced by optimizing the preparation method which highly influences the dispersion of the fillers within the sample. Approaches for improvements should therefore focus on increasing wear resistance and IL release into the wear trace under load by optimization of IL-filler interactions and ratios.

## 5 Conclusions and summary

In this work, carbon black (CB) or multi-walled carbon nanotubes (MWCNT) containing polypropylene (PP) with embedded [P_66614_][DOC] (IL) was characterized regarding self-lubricating, electrical conductivity and tribological properties. Self-lubricating polymer compounds could be identified depending on the tribological load (velocity, pressure). IL-Droplets were found in the bulk material for high IL contents with cold-fracture SEM micrographs, which also explain the mechanical weakening with increasing IL contents. The beneficial increase of IL in the wear trace and the weakening of the mechanical properties are directly influenced by the IL content. Testing parameters influence the amount of necessary IL in the wear trace to realize a stable tribofilm. Furthermore, we have shown fundamental differences between CB and MWCNT fillers in a PP matrix regarding their interaction with the IL and their tribological behavior. We received evidence for the interaction of phosphorous based cations in alkyl-phosphonium containing ILs with MWCNTs by ATR-FTIR spectroscopy, which leads to lower amount of IL in the wear track in comparison to CB based systems. Therefore, the amount of IL released in the wear trace depends on the interaction with the filler, thus key parameters for obtaining self-lubrication in a wide parameter range are high IL-contents without mechanical weakening of the matrix. The surface electrical resistance of PP compounds does not change significantly with the IL content, but the electrical resistance decreases with increasing filler content. In summary, the results presented in this work lead to a better understanding of polymeric, self-lubricating systems and will guide further developments in this area.

## Author contributions

A. K. and J. R. conceived and supervised the research. S. G, F. G. and T. A. designed and performed the experiments and analyzes. All authors discussed the results and wrote the manuscript. All authors reviewed the manuscript.

## Conflicts of interest

There are no conflicts to declare.

## Supplementary Material

RA-013-D3RA00712J-s001
