# Supplementary material for: Tribological performance of electrically conductive and self-lubricating polypropylene–ionic-liquid composites
Source: RSC Adv. 2023 Mar 10;13(12):8000–14. doi: 10.1039/d3ra00712j (PMC9999252; doi:10.1039/d3ra00712j)
Supplement: RA-013-D3RA00712J-s001 [file RA-013-D3RA00712J-s001.pdf]

Electronic Supplementary Material (ESI) for RSC Advances.
This journal is © The Royal Society of Chemistry 2023

ESI

1. Detailed information on geometries and parameters used

Table S 1: overview on chosen parameters for measurement series.

Geometry	Load / N	Reciprocatin g length l / mm	Frequ ency v / Hz	Material pairing (oscillating / stationary	Conta ct area / mm^2	Initial contact pressure p/ $N \cdot mm^{-2}$	v_{max}[a] / $m \cdot s^{-1}$	$p \cdot v_{max}$ / $Nm \cdot (s \cdot mm^2)^{-1}$
Ball on disk	10	1	20	100Cr6 / PP-compound	0.297	50.469	0.063	3.171
Cylinder on disk	10	1	50	100Cr6 / PP-compound	0.058	7.226	0.157	1.135

[a] calculated by the following formular: $v_{max} = \pi\, l \cdot v$

1. Sample preparation and experiment

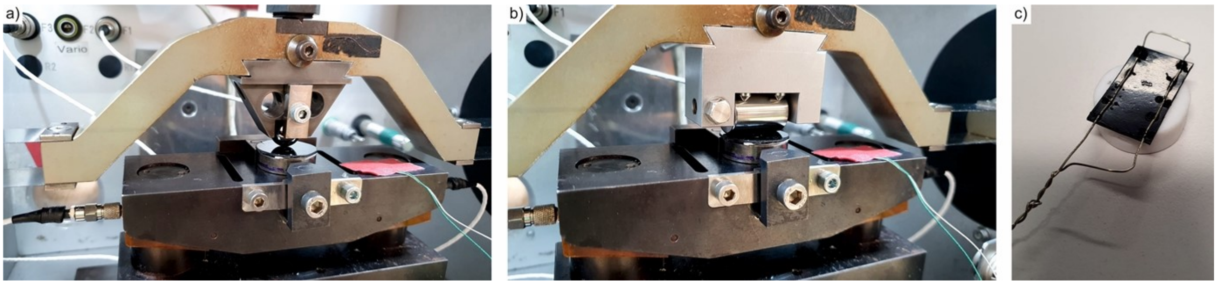

Figure S 1: Sample fixed on friction and wear testing machine SRV IV (Co. Optimol Instruments Prüftechnik GmbH) with a) a sphere on disk geometry and b) a cylinder on disk geometry. c) Measured sample with applied potential.

2. SEM and EDX analysis of pristine samples and their wear traces

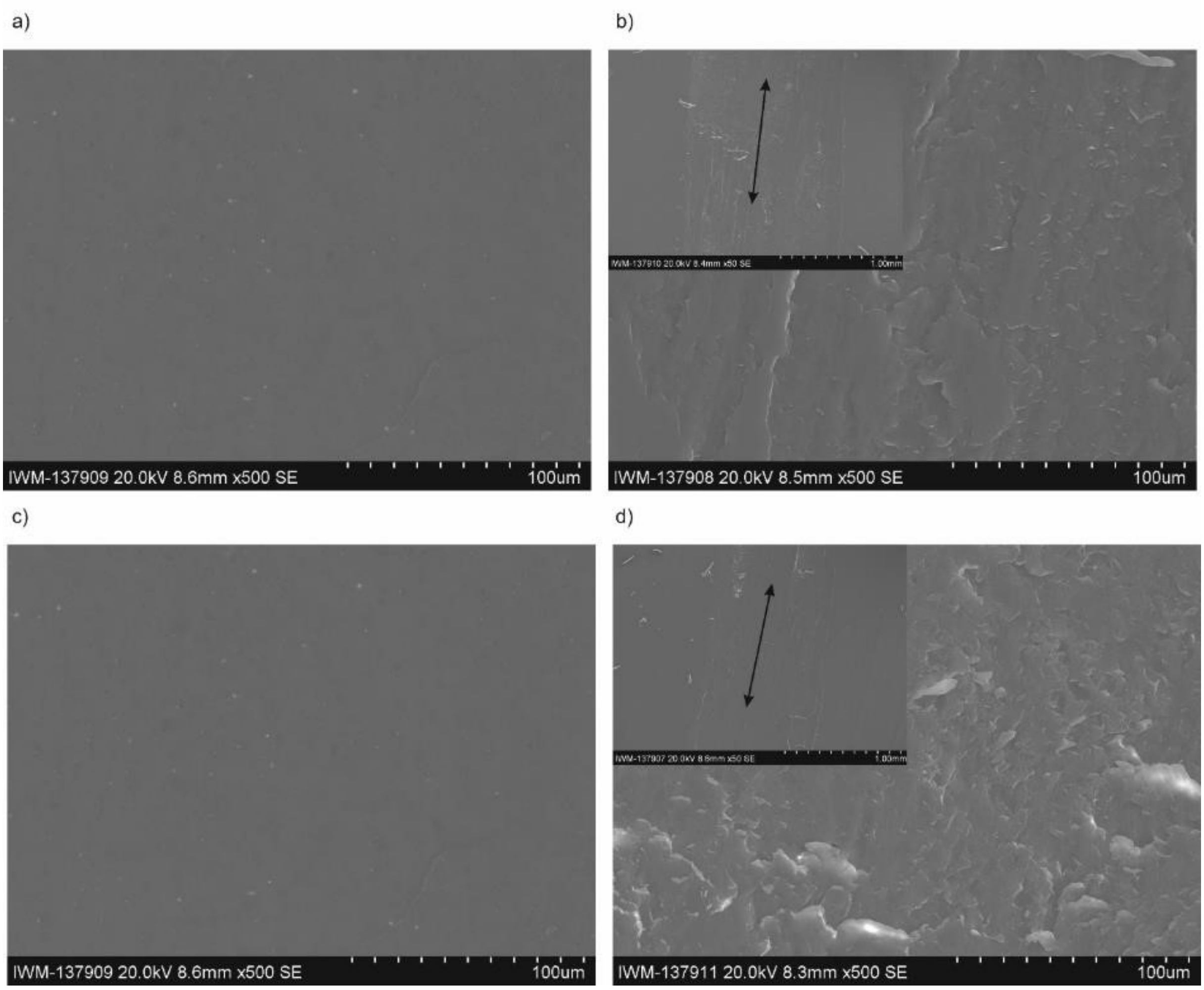

Figure S2: a) SEM-picture of the C-3 compound as obtained from the kneader on the surface and b) in the wear trace. c) SEM-picture of the surface of the same compound as obtained from the extruder by injection molding and d) from its wear trace.

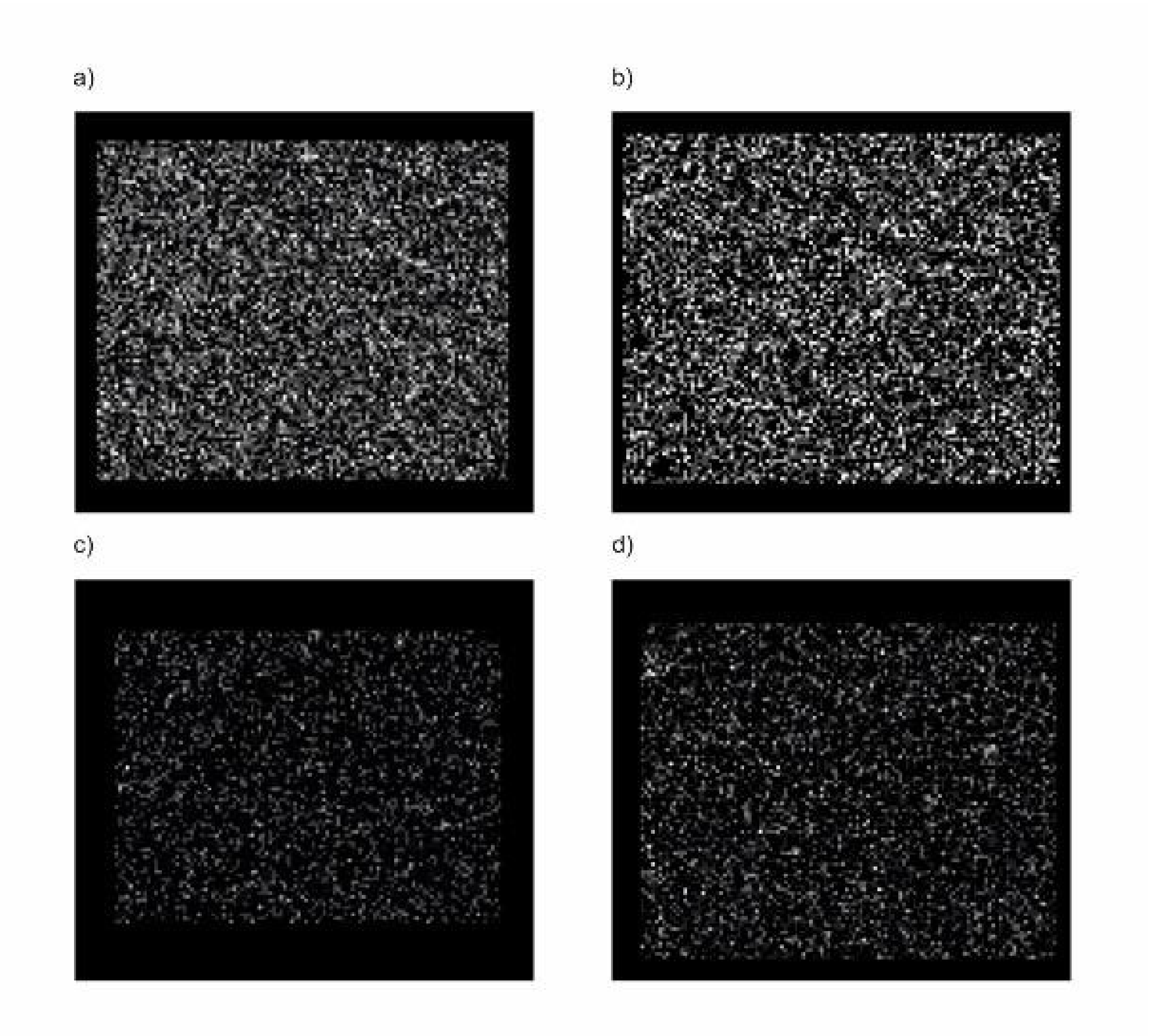

Figure S3: a) EDX-map from the compound C-3 as obtained from the kneader on the surface and b) in the wear trace. c) EDX-map of the surface of the same compound as obtained from the extruder and d) from its wear trace.

As shown in Figure S *2* SEM-pictures were recorded for C-3 IL as obtained from the kneader (pictures a) and b)) and from the extruder by injection molding (pictures c) and d)). For both preparation methods smooth surfaces are obtained, as shown in pictures a) and c). However, when it comes to wear traces the surface of the compound as obtained from the extruder by injection molding seems to consist of smaller agglomerated and sheet-like particles in comparison to the same compound as obtained from the kneader. The associated phosphor EDX-maps are shown in Figure S 3. Whereas the wear trace and the surface for samples prepared by a kneader are similar to each other the wear trace of extruder samples shows higher phosphor content than the bare surface (picutres c) and d)), as visualized by different densities of bright spots. Also the extruder samples show lower surface concentrations of phosphor than the kneader samples, as shown in pictures a) and c) in Figure S 3. This might explain the higher electrical surface resistance of samples obtained by injection molding as the IL and therefore phosphorous should be mainly located in CNT domains and not in PP domains. For both samples the phosphor distribution is nearly homogenous with small local deviations.

3. Data for tribological testing, electrical surface resistance and Vickers hardness (HV)

Table S 2: COF with different parameter sets, electrical surface resistance and Vickers hardness (HV) for all compounds.

PP-content / wt. %	MWCNT-content / wt. %	IL-content / wt. %	Abbreviation	Electrical surface resistance / $\Omega \cdot$ cm	HV	COF (50 Hz, cylinder on disk geometry)	COF (20 Hz, sphere on disk geometry)	COF (50 Hz, sphere on disk geometry)
100.0	0.0	0.0	A-0[a]	$>1 \cdot 10^6$	12.4 ± 0.3	0.86 ± 0.06	0.47 ± 0.05	0.50 ± 0.06
99.0	0.0	1.0	A-1	$>1 \cdot 10^6$	12.0 ± 0.3	0.73 ± 0.09	0.59 ± 0.05	-
98.0	0.0	2.0	A-2	$>1 \cdot 10^6$	11.3 ± 0.5	0.79 ± 0.13	0.66 ± 0.04	-
97.5	0.0	2.5	A-3	$>1 \cdot 10^6$	12.4 ± 0.3	0.72 ± 0.07	0.51 ± 0.05	-
99.0	1.0	0.0	B-0	237 ± 37	15.1 ± 0.5	0.90 ± 0.05	0.61 ± 0.06	-
98.0	1.0	1.0	B-1	135 ± 15	15.5 ± 0.5	0.77 ± 0.04	0.58 ± 0.03	-
97.0	1.0	2.0	B-2	182 ± 22	13.5 ± 0.6	0.72 ± 0.07	0.620 ± 0.009	-
97.6	2.4	0.0	C-0	11.7 ± 0.9	16.0 ± 0.2	0.65 ± 0.07	0.65 ± 0.07	-
96.6	2.4	1.0	C-1	8.6 ± 0.6	15.3 ± 0.8	0.72 ± 0.09	0.60 ± 0.02	-
95.6	2.4	2.0	C-2	8.1 ± 0.4	14.4 ± 0.4	0.79 ± 0.11	0.61 ± 0.08	-
95.1	2.4	2.5	C-3[a]	$>1 \cdot 10^6$	13.6 ± 0.5	0.75 ± 0.10	0.60 ± 0.04	-

PP-content / wt. %	CB-content / wt. %	IL-content / wt. %	Abbreviation	Electrical surface resistance / $\Omega \cdot$ cm	HV	COF (50 Hz, cylinder on disk geometry)	COF (20 Hz, sphere on disk geometry)	COF (50 Hz, sphere on disk geometry)
99.0	1.0	0.0	D-0	$>1 \cdot 10^6$	16.0 ± 0.2	0.65 ± 0.02	0.51 ± 0.07	-
98.0	1.0	1.0	D-1	$>1 \cdot 10^6$	13.5 ± 0.2	0.70 ± 0.04	0.56 ± 0.05	-
97.6	2.4	0.0	E-0	$>1 \cdot 10^6$	15.3 ± 0.3	0.66 ± 0.04	0.61 ± 0.04	-
95.1	2.4	2.5	E-3	611000	16.0 ± 0.5	0.76 ± 0.03	0.59 ± 0.02	-
95.0	5.0	0.0	F-0	691	15.7 ± 0.3	0.73 ± 0.06	0.53 ± 0.04	-
90.0	5.0	5.0	F-5	118	14.2 ± 0.2	0.65 ± 0.04	0.6431 ± 0.0009	1.09 ± 0.02
85.0	5.0	10.0	F-10	332	10.7 ± 0.2	0.74 ± 0.09	0.64 ± 0.03	-
80.0	5.0	15.0	F-15	235	8.4 ± 0.2	1.38 ± 0.06	0.93 ± 0.04	-

[a] This sample has been prepared by different preparation methods, whereas the sample prepared by an extruder and injection molding is denoted as C-3e and A-0e.

4. Optical- and height images after testing with a ball on disk geometry

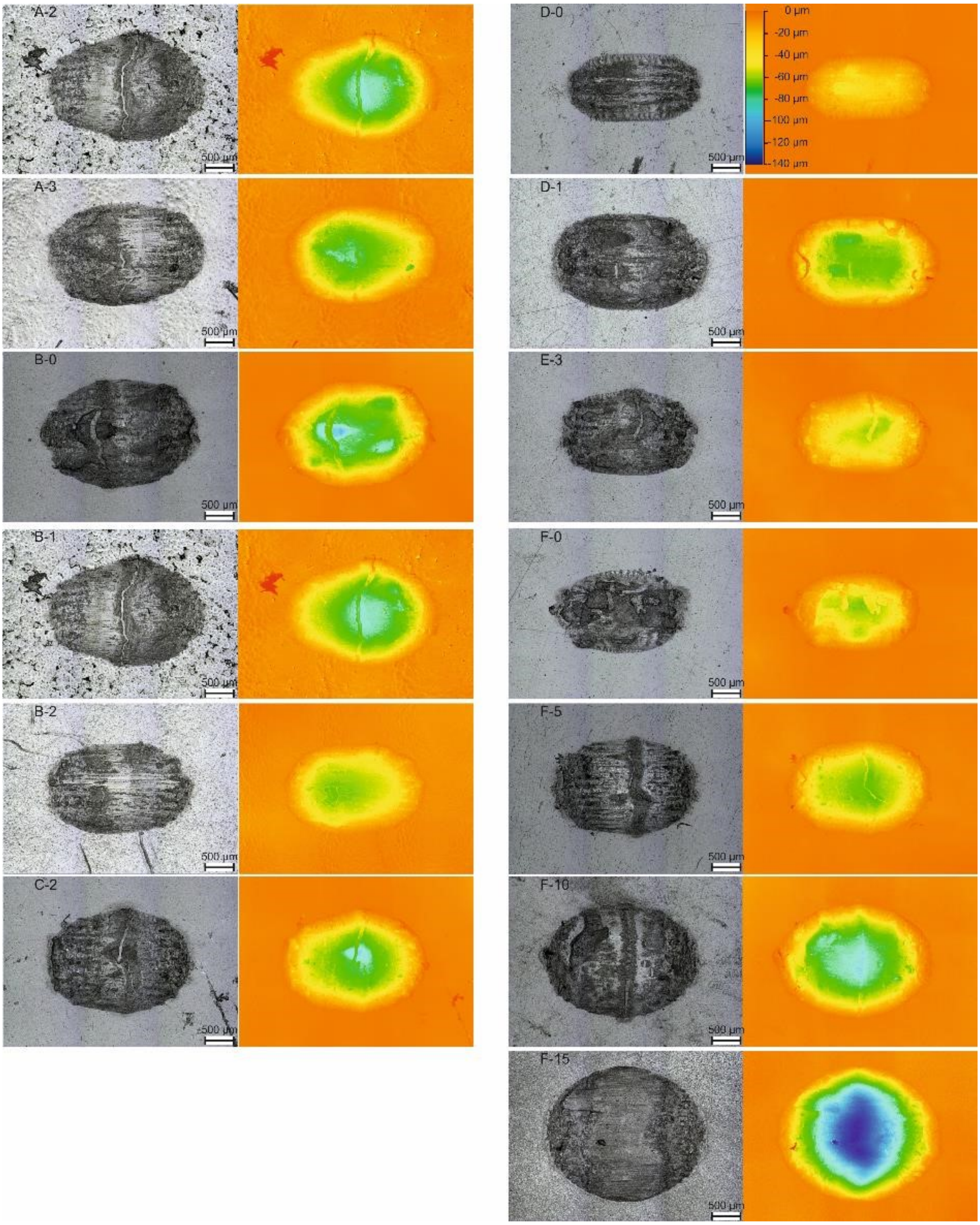

Figure S4: Optical- and height images of all samples.

5. Measured and simulated IR-spectra of [P_{66614}][DOC]

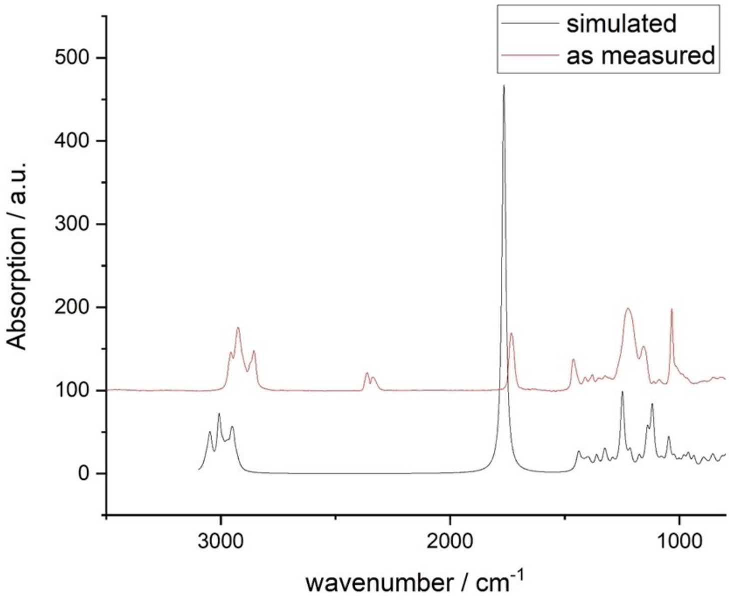

Figure S5: Simulated and measured IR spectra of [P_{66614}][DOC]. Simulated spectra fitted with Lorentzian type functions. Shift to higher frequencies due to Rayleigh-Ritz method. See [70].

6. Influence of preparation techniques

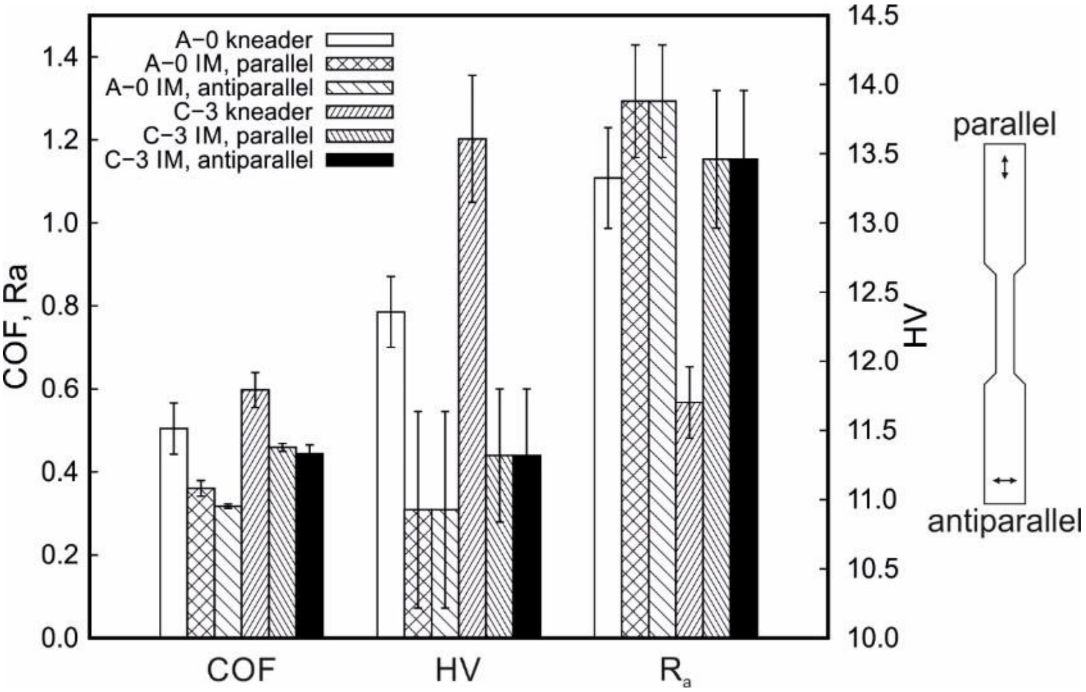

Figure S6: COF, HV and R_a for A-0 and C-3 compound as prepared by a kneader in comparison with the preparation via injection molding. COF for A-0 compound was measured with a ball on disk geometry with 10 N of load and a reciprocating frequency of 50 Hz. COF for C-3 compound was measured with a ball on disk geometry with 10 N of load and a reciprocating frequency of 20 Hz. This equals a reduction of 37 % (A-0) and 25 % (C-3) in COF. Other than that, the electrical surface resistance was influenced as well and increased from a value of 10(1) $\Omega \cdot$ cm for the C-3 compound to a value higher than $1 \cdot 10^6 \Omega \cdot$ cm.
